# Sustainable Use of Volcanic Ash in Mortars as a Replacement for Cement or Sand: Shrinkage and Physical and Mechanical Properties

**DOI:** 10.3390/ma18153694

**Published:** 2025-08-06

**Authors:** Luisa María Gil-Martín, Miguel José Oliveira, Manuel Alejandro Fernández-Ruiz, Fernando G. Branco, Enrique Hernández-Montes

**Affiliations:** 1 Department of Structural Mechanics, University of Granada (UGR), Campus Universitario de Fuentenueva s/n, 18072 Granada, Spain; mlgil@ugr.es (L.M.G.-M.); emontes@ugr.es (E.H.-M.); 2 Departamento de Engenharia Civil (DEC), Universidade do Algarve, Faro and CERIS, 8005-139 Faro, Portugal; mjolivei@ualg.pt; 3 Departamento de Ingeniería Civil, de Materiales y Fabricación, Universidad de Málaga, C/Dr. Ortiz Ramos, s/n, 29071 Málaga, Spain; 4 Department of Civil Engineering, University of Coimbra, ISISE, ARISE, 3030-790 Coimbra, Portugal; fjbranco@dec.uc.pt

**Keywords:** mortar, volcanic ash, physical–mechanical properties, shrinkage

## Abstract

The eruption of the Cumbre Vieja volcano on 19 September 2021 resulted in the deposition of over 20 million cubic meters of tephra, posing significant environmental and logistical challenges in the affected areas. This study aimed to explore the valorization of volcanic ash (VA) by evaluating its potential use in producing sustainable mortar by incorporating it as a replacement for cement or sand. Various experimental mixtures were prepared with different proportions of VA which substituted either cement or sand, and these mixes were characterized through a mechanical and microstructural campaign. Additionally, shrinkage was evaluated for the mixtures which showed good mechanical results. The results suggest that partially replacing cement with up to 15% ground VA as well as substituting sand with up to 25% VA are promising strategies for the production of sustainable mortar mixes. This research contributes to the understanding of the influence of VA in cementitious matrices and offers a novel approach for integrating locally available geomaterials into infrastructure design in volcanic active regions.

## 1. Introduction

The island of La Palma (Spain) has experienced considerable volcanic activity over the last few centuries. The Cumbre Vieja volcano is the most active in the Canary Islands, having erupted eight times between 1500 and 2020 [1,2]. The recent eruption of the Cumbre Vieja volcano in 2021 was the longest in its recorded history [3], and millions of cubic meters of pyroclastic ash and coarser particles were scattered across the surface of La Palma [4,5].

One of the primary tasks initiated by the government after the eruption was the removal of volcanic ash (VA), followed by transporting it to landfill sites, where it was deposited. If VA is not removed, it can pose potential hazards to human health, as it can cause respiratory problems and/or eye and skin irritations [6,7]. Currently, VA is disposed of in landfills, and there is no clear legislation regarding its disposal and recycling [8]. Hence, the reuse of VA has emerged as a significant objective in both municipal strategies and engineering scientific studies.

Utilizing volcanic products in various construction applications can be an effective way of valorizing them, promoting the sustainable use of this natural material and reducing the carbon footprint of the construction industry [9]. Pyroclastic materials have been evaluated for their potential use in various fields of the construction sector, including bricks [8], self-compacting concrete [10,11], the reconstruction of new road infrastructures [12], and mortars [13,14,15].

The heterogeneity of the physical and mechanical properties of volcanic products necessitates mechanical characterization in each specific case. The chemical composition of La Palma VA allows it to be considered as a material with pozzolanic properties [3,16,17], making it a candidate for use in mortars as a partial substitute for cement. In [3,16], the percentages of cement replacement with VA ranged between 10% and 40%. In [18], the percentage of cement replacement with VA was increased, reaching complete replacement (i.e., 0% cement). In general, a maximum replacement level of 20% for Portland cement is recommended, as higher dosages tend to result in a notable reduction in mechanical performance compared to reference mixes [19]. Other authors have investigated alternative cements by using Cumbre Vieja VA [20,21]. The rapid development of compressive strength reported in [20] encourages the use of VA for prefabricated elements in the reconstruction efforts on La Palma Island. In [22], volcanic ash was employed as a raw material for the synthesis of inorganic polymers (geopolymers), utilizing only sodium hydroxide as the alkaline activator.

Aligned with the principles of the circular economy, this study explores the valorization of a waste product (Cumbre Vieja VA), thereby reducing the carbon footprint of the concrete industry. For this purpose, VA was employed as a substitute for both cement and sand in mortars. Due to rapid cooling, VA is characterized by a high content of vitreous phases and is mainly constituted by silica and alumina, which together account for at least 70% of its weight [19], endowing it with pozzolanic activity. Additionally, it contains appreciable levels of Fe_2_O_3_ and MgO [23]. Various review studies explored how the chemical and mineralogical features of VFAs, as well as their reactivity, are influenced by their origin, considering both the geographical setting and the type of volcanic activity [9,19]. It is well known that the properties of mortars incorporating VA as cement substitute depend on both the content of VA and its fineness [24]. The closer the fineness of the VA is to that of cement, the more effective the hydraulic reactions between them will be [16]. In [3,18], VA was ground to particle sizes below 45 microns (45 μm), while in [16], the fineness of the VA was even higher than that of the cement. Consequently, in this work VA was ground until 96% of the material passed through a 425 μm sieve, and three levels of cement replacement were investigated: 5%, 10%, and 15%. Additionally, raw VA (as collected on-site) was used as a substitute for sand at replacement levels of 12.5%, 25%, 50%, and 75%. Notably, the use of VA as a sand substitute in mortar formulations has received limited attention in the existing literature. The mortars studied in this work do not have a specific application but are intended for general use, similar to conventional mortar.

Mechanical and physical properties of the mortar mixtures, such as compressive and flexural strength, flowability, bulk density, water absorption, and porosity, were determined. In addition, shrinkage (caused by both air and immersion) was evaluated for the mixtures that showed good mechanical results. To the authors’ knowledge, the effect of replacing cement or sand with La Palma VA on the shrinkage of mortars has not been mentioned in the literature. Moreover, as many factors can vary the chemical composition and mineralogy of volcanic ash, the VA used in this study was characterized using X-ray fluorescence (XRF) and X-ray diffraction (XRD). Finally, the morphology and microstructure of some types of mortar were analyzed using scanning electron microscopy (SEM).

## 2. Materials and Methods

### 2.1. Raw Materials

**Figure 1:**
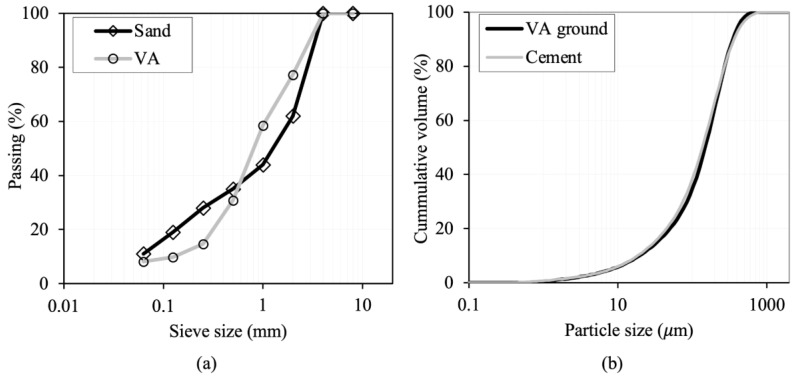
Sand and VA grading (**a**) and cement and ground VA particle size distribution (**b**).

A dolomitic crushed sand was used in this work (the corresponding grain size distribution is shown in Figure 1a). In previous studies [18,25], VA from the Cumbre Vieja volcano was used as a partial replacement for cement in mortar production. These mortars were produced using ordinary Portland cement (OPC). However, growing environmental concerns have led many construction standards to recommend the use of Portland-composite cement (CEM II) as a more sustainable alternative to reduce CO_2_ emissions. Hence, cement CEM II/B-L 32.5 N (SO_3_ 2.9%, Cl 0.01%) was used. This type of cement is widely employed in construction, particularly in hot climates, due to its slower setting time, which helps mitigate the rapid setting often encountered under such conditions. Figure 1b shows the particle size distribution (PSD) of the cement obtained with laser diffraction (LD) using a MASTERSIZER 2000LF analyzer manufactured by Malvern Panalytical (Malvern, UK). The measurements were conducted in wet mode with water as the dispersant medium, and the Hydro 2000 G accessory was employed to ensure proper particle dispersion during analysis.

As a partial substitute for sand, VA was used in its original state as collected on-site (see grading curve in Figure 1a). For its use as a partial substitute for cement, VA was ground by placing it into the sealed stainless-steel bowl of the Micro-Deval testing equipment with an abrasive charge consisting of 9.5 mm diameter stainless-steel balls for a total of 4500 revolutions. At the end of the abrasion test, 96% of the material passed through the 425 μm sieve (see PSD of ground VA in Figure 1b).

The chemical composition of VA, sand and cement was obtained using XRF with a Zetium spectrometer model, manufactured by Malvern Panalytical (Malvern, UK), which is a 4 kW device. The mineralogical analysis of the VA was conducted using powder XRD on a BRUKER D8 ADVANCE device manufactured by Bruker (Karlsruhe, Germany) and equipped with a Cu anode. Finally, the apparent particle density of ground VA was determined using the pycnometer method in accordance with EN 1097-6 [26].

XRF analyses of VA indicated a predominantly siliceous composition, with a SiO_2_ content of 43.63% (see Table 1). Additionally, significant amounts of Al_2_O_3_ (13.47%), Fe_2_O_3_ (13.3%), CaO (11.27%), and MgO (8.39%) were present. The combined total of silicon, aluminum, and iron oxides is 70.33%, which exceeds the minimum requirement of 70% set by the US ASTM C618 standard [27] for N- and F-type additions, and the VA with a CaO content of 11.27% could even have cementing properties. Similar results were obtained in [3], where VA from La Palma was studied as a Portland cement constituent. In Table 1, LLD refers to the lower limit of detection, defined as the smallest concentration of an element that can be reliably detected above the background noise.

**Figure 2:**
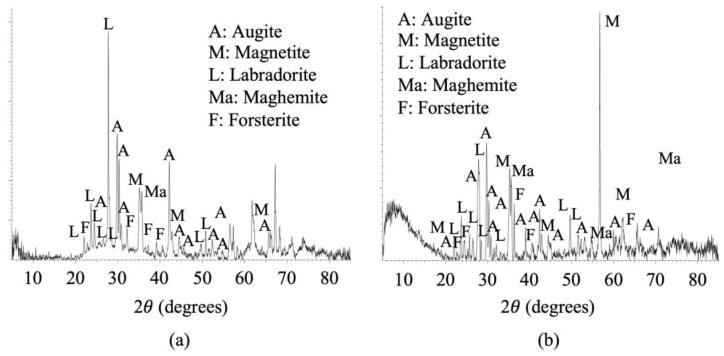
Mineralogical analysis of sieved VA (**a**), and ground VA (**b**) using XRD. EVA V7 software cards employed: 9,009,664 augite, 9,013,535 magnetite, 9,000,748 labradorite, 9,006,316 maghemite, and 9,000,314 forsterite.

A mineralogical analysis of VA using XRD, in both its original (the finest portion) and ground states (see Figure 2a and Figure 2b, respectively), showed a substantial presence of ferromagnesian minerals. The measurements were carried out over a 2θ range from 5° to 85°, with a step size of 0.02° and a counting time of 40 s per step.

The average apparent particle density of VA was 2802 ± 130 kg/m^3^. This result is consistent with the findings reported by Jubera-Perez et al. [17], in which various VA samples from La Palma were studied.

### 2.2. Mixture Proportions and Mixing Procedure

Mortar mixtures were prepared as prescribed by EN 196-1 [28]. Hence, the proportions by mass of the reference mixture were one part cement, three parts sand, and half a part water. In this work, three types of mixtures were compared: traditional or reference mortar (RM), mortar incorporating ground VA as a cement replacement (VA-C), and mortar incorporating VA as a sand replacement (VA-S). The replacement percentages are specified in the sample labels: VA-C-5%, VA-C-10%, and VA-C-15% for VA-C mortars, and VA-S-12.5%, VA-S-25%, VA-S-50%, and VA-S-75% for VA-S mortars. Table 2 summarizes the dosages of the mortar mixes studied in this work.

In the last two mixtures (see Table 2), the SIKA ViscoCrete-6003^®^ NG superplasticizer (Sika, Baar, Switzerland) was added to improve workability. The dosages used were within the range recommended by the supplier (0.4 to 1.8% of the weight of the cement).

The mortar mixture was transferred directly from the bowl into the molds in two layers. The molds consisted of three horizontal compartments, allowing for the simultaneous preparation of three prismatic specimens, each of which was 40 mm × 40 mm × 160 mm. Each batch of three specimens was comprised of the material quantities shown in Table 1. After 24 h, the specimens were demolded and submerged in water at 20 ± 1 °C until testing.

### 2.3. Tests Performed

Tests to characterize the mechanical, physical, and chemical parameters of all the mortar mixtures studied in this work were carried out.

**Figure 3:**
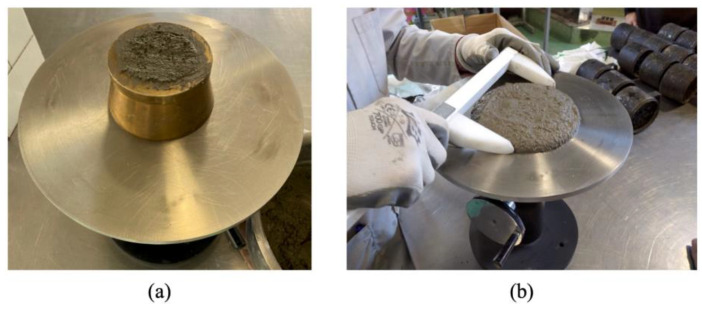
Flow test. Truncated mold (**a**) and measurement of the diameter of the mortar (**b**).

The bulk densities of both fresh and hardened mortars were determined. The flowability of all the mortar mixtures was evaluated using a flow table in accordance with EN 1015-3 [29] (see Figure 3a,b). The average experimental data of three samples were recorded.

**Figure 4:**
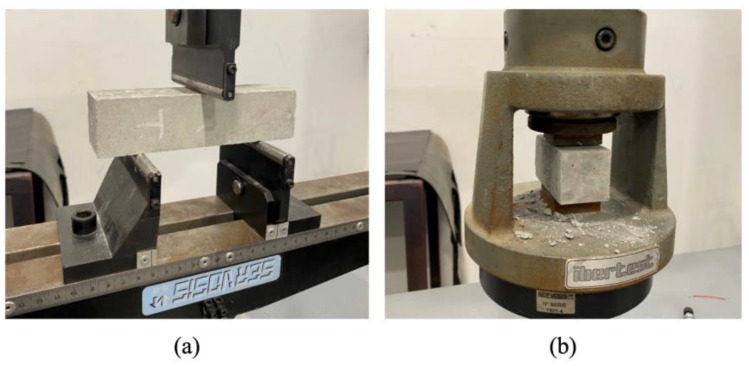
Flexural (**a**) and compression (**b**) test setups.

The flexural strength of the mortar mixtures was determined in accordance with EN 1015-11 [30]. Three prismatic specimens (40 mm × 40 mm × 160 mm) were tested after each mortar mixture had been cured for 28, 90, and 200 days. The flexural tests were conducted using a universal testing machine (UTM) with a maximum load of 100 kN (see Figure 4a). The span in the tests was 100 mm. During the tests, the load was increased in displacement control at a rate of 0.3 mm/s.

The compressive strengths of all the mortar mixtures studied in this work were determined on the two parts resulting from the flexural strength test in accordance with EN 1015-11 [30]. Hence, six specimens were tested after being cured for 28, 90, and 200 days, after the flexural tests. The compression tests were conducted using a UTM with a maximum load of 100 kN (see Figure 3b). The load was applied to the faces of the specimens that were cast against the steel of the mold. During the tests, the load was increased in displacement control at a rate of 1 mm/s. Compressive and flexural tests were conducted first in the experimental campaign. At this stage, the VA-S-75% mortar mixture was excluded from the study due to its low flexural and compressive strengths.

One of the primary pathways for aggressive substances to infiltrate construction materials is water absorption. Therefore, the performance of the mortar mixes in the presence of water was evaluated by quantifying two parameters: water absorption after 48 h of immersion [31] and water absorption via capillarity [32].

The water absorption tests were carried out on 56 days old 15 mm × 15 mm × 50 mm mortar specimens. The specimens underwent a preconditioning procedure which involved drying them in an oven at 60 ± 5 °C until a constant mass was achieved. The samples were then placed in a desiccator and allowed to cool before being weighed to obtain their dried mass. The immersion test involved fully submerging the specimens in a hermetic box filled with water for 48 h. After the 48 h period, the specimens were removed from the box and cleaned using a damp cloth to obtain the saturated mass of each sample (*m*_1_). The water content at 48 h (*W*_48h_) is expressed as a percentage and determined by the following equation:

(1) $$W_{48h}=\frac{m_{0}-m_{1}}{m_{1}}\times 100\;[\%]$$

In Equation (1) *m*_0_ represents the mass of the specimen in the dried state, and *m_1_* is the specimen mass after 48 h immersion. The capillary water absorption coefficient and the capillary water penetration coefficient of each mixture studied were determined in accordance with [32]. Before the test, the specimens were preconditioned in a similar way to those used for the water immersion test, and their dry mass (*m*_0_) was recorded. A dry bedding layer with a minimum thickness of 5 mm was placed on the bottom of the test vessel. Water was added until the bedding layer reached saturation. Special care was taken to ensure that the water level did not exceed the upper surface of the bedding layer, and the water level was kept constant throughout the test by adding water as necessary. At the beginning of the test, each pre-conditioned specimen was weighed (*m*_2_). The surface under investigation was placed on the bedding layer, and the chronometer was started. At prescribed moments along time, the water absorption on the specimen was evaluated. Mass evaluations, as well as the height of the damp mark on the lateral surfaces of each specimen, were computed and recorded. The specimen was taken off the medium, any water adhering to the surface was wiped off using a damp cloth, and the specimen was weighed (*m_i_*). Then, the maximum observed height of the damp mark on the lateral surfaces was measured and recorded, and the specimen was returned to the vessel to continue the capillarity absorption process. The capillarity absorption coefficient of each specimen was then computed according to the test procedure proposed in [32]. The amount of water absorbed by the specimen per unit area *Q_i_* (kg/m^2^) at time *t_i_* (s) is calculated by Equation (2):

(2) $$Q_{i}=\frac{m_{i}-m_{2}}{A}$$

where *m_i_* is the mass of the specimen at time *i* (s), and *m*_2_ is the mass at the start of the test (dry state).

For the determination of the capillary water absorption curve the calculated values of *Q_i_* are reported in a graph as a function of the square root of time (*t_i_*^1/2^). The capillary water absorption coefficient (*AC*) is the slope of the linear section of the curve obtained plotting the mass change per area (*Q_i_*) versus the square root of time (*t_i_*^1/2^), and is calculated by linear regression, using at least 5 successive aligned points. The capillary water pene-tration coefficient *B* is represented by the slope of the curve obtained reporting the height of the water front migration (*H_i_*) versus the square root of time (*t_i_*^1/2^) and is also calculated by linear regression.

Mercury intrusion porosimetry [33] was used to analyze the macroporosity of the mortar mixtures. The porosimetry test was performed with an AutoPore IV 9500 porosimeter model, manufactured by Micromeritics, Norcross, GA, USA. The specimens were dried at 50 °C for 48 h prior to the test. In this research, the total porosity and pore size distributions of the mortars were analyzed. The pore size distributions were represented using the cumulative volume of pores (in percentage) versus pore size curves.

Three mortar mixtures were selected for the shrinkage tests: RM, VA-C-15%, and VA-S-50%, with replacements that fall within the typical ranges considered in the literature [3,16,34]. The selection was based on achieving a balance between flexural and compressive strength values, as well as the VA content. Since the aim of the study was to quantify the influence of VA on shrinkage, among all the samples, those with the highest ash content that did not exhibit an unacceptable reduction in strength were selected. The preparation of three specimens (40 mm × 40 mm × 160 mm) of each mixture was performed in accordance with EN 196-1 [28], in a room with a temperature of 20 ± 2 °C and a relative humidity of 55 ± 5%. The molds were removed about 24 h after mixing. This is the minimum period of time required to ensure that the mortar is strong enough to prevent damage to the specimens during the removal of the molds, which is estimated to be between 3 and 5 MPa. Subsequently, the specimens were weighed, and their lengths were recorded. After the molds were removed, for comparison purposes, two curing levels were specified: uncured (air curing at a temperature of 20 ± 2 °C and a relative humidity of 55 ± 5%) and immersed (in water at a temperature of 20 ± 2 °C). The intention was to evaluate the performance under different conditions of possible use: (1) in a drier environment where shrinkage is subject to certain standardized drying conditions; (2) in a submerged environment where the conditions are opposite and in which eventually expansions can be recorded [35]. Three specimens were tested for each curing level. Shrinkage deformations of each specimen were measured using a length comparator with a sensitivity of 1 µm and gage studs on the end sections of the mortar prisms. The stability of the length comparator was checked using a reference invar bar. Samples were weighed and measured at 1, 3, 7, and 14 days, and at 1, 2, 3, and 4 months.

Finally, to observe the morphology of the specimens, a high-resolution field-emission scanning electron microscope, AURIGA (FIB-FESEM) Carl Zeiss SMT (Carl Zeiss AG, Oberkochen, Germany), was used. SEM specimens (slices of 15 mm × 15 mm with a thickness of 30 μm) were carbon-coated using a Polaron CC7650 device (KLA Corporation, Milpitas, CA, USA). The mixtures scanned were as follows: RM, VA-C-10%, VA-C-15%, VA-S-25%, and VA-S-50%, corresponding to the mixes with the best compressive strengths and those whose shrinkages had been studied. Electron dispersive spectroscopy (EDS) analysis was employed to discern the fundamental elements of the mortar mixes.

## 3. Results and Discussion

### 3.1. Mechanical Strength

**Figure 5:**
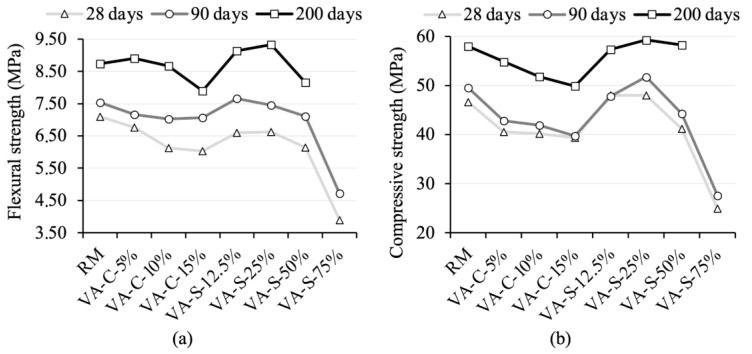
Flexural (**a**) and compressive (**b**) strengths of the mortar mixtures after being cured for 28, 90, and 200 days.

Figure 5 shows the compressive strength and the flexural strength of all the mortar mixes after being cured for 28, 90, and 200 days. As shown in Figure 5b, the compressive strength of VA-C samples at 28 days is lower than that of RM. This trend continued after being cured for 90 and 200 days. The mortar’s strength decreases as the VA content increases. This behavior is expected, as the pozzolanic activity of the ash is lower at early ages, leading to delayed cement hydration with higher ash content [3]. Conversely, the VA-S-12.5% and VA-S-25% mixtures exhibited higher compressive strengths than RM at 28, 90, and 200 days. The increase in compressive and flexural strength attributed to VA was due to the dominance of silica-based material (SiO_2_), which proved to be an effective filling agent [34]. The empty cavities within the cement particles were filled with silica, enhancing its mechanical properties and durability. Conversely, the decrease in compressive and flexural strength in VA-S-50% was caused by an excessive proportion of volcanic ash, leading to a non-homogeneous mixture. The VA-S-75% mixture, however, showed very low compressive strength as a result of its high porosity, which was visibly apparent. As a result, this mixture was excluded from the study.

A similar pattern can be observed with flexural strength. The VA-C samples showed lower values than RM, while the VA-S samples (up to 25% replacement) showed higher flexural strength. Figure 5b shows that, at 200 days, the flexural strengths of the VA-C-5% and VA-C-10% mixtures are within the range of that of the RM, and the strength of the smaller cement replacement is even slightly higher.

Both flexural and compressive strengths increase with longer curing times. Figure 5 shows that, compared to RM, the studied mixtures show a more significant increase in flexural and compressive strength between 28 and 200 days. The exceptions are the VA-S-12.5% and VA-S-25% mixtures, in which the compressive strength gain is slightly lower than that of the RM between 28 and 200 days. These results can be interpreted in the light of a possible pozzolanic reaction between VA and portlandite, produced during cement hydration, as reported in [3]. It is also worth noting that the outcomes might have differed if a pure OPC had been used. In this study, a CEM II/B-L cement was employed, in which the amount of portlandite available for pozzolanic reactions was significantly lower than in a hydrated CEM I paste.

The results obtained in this study are consistent with the findings presented in [36], in which it was concluded that compressive strength decreases as the amount of cement replaced by VA increases. In [36], strength decreased by 9.5% at 28 days when the VA content was increased from 0% to 20%.

### 3.2. Shrinkage

**Figure 6:**
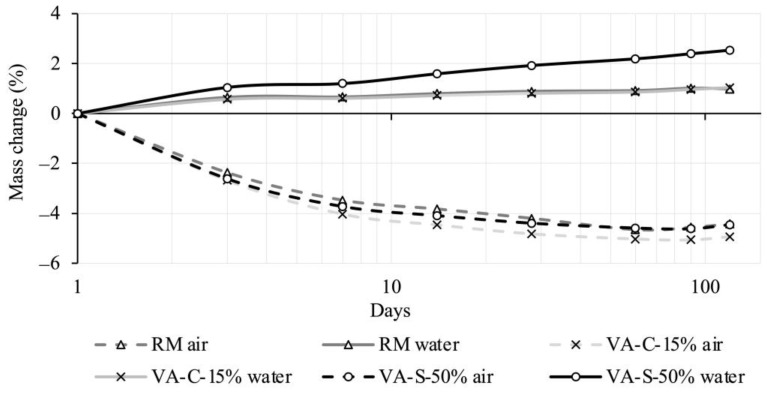
Mass change of RM, VA-C-15%, and VA-S-50% mixtures.

**Figure 7:**
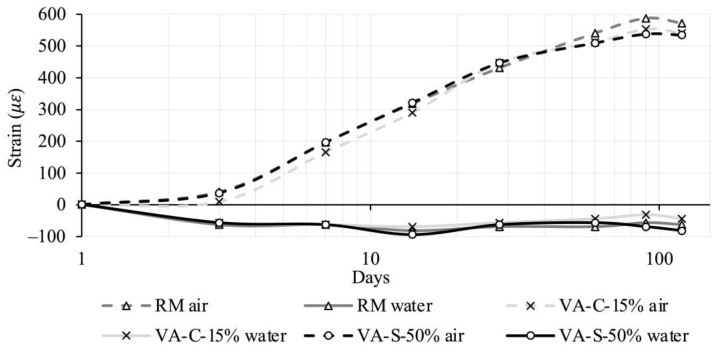
Total shrinkage evolution for the RM, VA-C-15%, and VA-S-50% mixtures, both in air-cured and immersed conditions.

Figure 6 and Figure 7 present the results of the shrinkage study conducted on the RM, VA-C-15%, and VA-S-50% mixtures. The chart in Figure 6 shows the mass variation for the mixtures recorded over a period of up to 4 months, both under air curing and immersed conditions. Each value presented is the average of three specimens. The deviation in the individual results was minimal (standard deviation lower than 0.16%). The mass variation is expressed as a percentage of the initial mass, which was recorded immediately after the removal from the mold.

**Figure 8:**
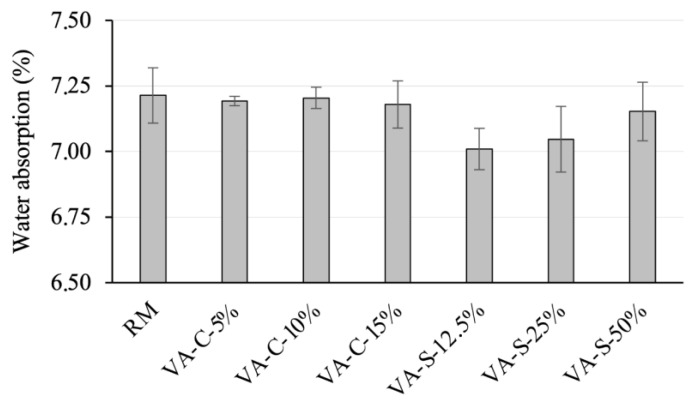
Water absorption levels of the mortar mixes.

Regarding mass variation, the mixtures generally followed the trend of the RM mixture, with the exception of the VA-S-50% mixture. In the immersed condition, this mixture showed a significantly higher level of water absorption after 4 months, which is in agreement with the results in Figure 8.

Figure 7 shows the total shrinkage evolution recorded over a period of up to 4 months for the RM, VA-C-15%, and VA-S-50% mixtures, both in air-cured and immersed conditions. As before, the results, presented in the form of a graph, were obtained using the average measurements of three specimens per mixture. The maximum standard deviation recorded was 26 microstrains, which is why the standard deviation is not represented on the graph.

Regarding the behavior of the air-cured specimens, there were no significant deviations compared to the reference mixture. The values observed are typical for a mortar with the maximum aggregate size used. A similar trend can be observed in the immersed specimens, in which the values followed the behavior of the reference mixture. In this case, the expansion values stabilized after 10 days, which may be advantageous in certain application situations.

### 3.3. Physical Properties of Mortar Mixes

#### 3.3.1. Bulk Density and Flowability

Table 3 presents the bulk density of both fresh and hardened mortars, as well as the slump flow diameter of the mixtures. It shows that the bulk density of the reference mortar is higher than the other mixes studied. Generally, bulk density decreases as the percentage of cement or sand replaced with VA or ground VA increases.

Slump flow measurements for the different mixtures showed a decrease as the proportion of sand replaced by VA increased. In contrast, mortar mixtures with cement substituted by VA exhibited a slightly higher slump flow than RM. Mortar mixtures—RM, VA-C-5%, VA-C-10%, VA-C-15%, and VA-S-12.5%—can be categorized as plastic mortars (with slumps ranging between 140 and 200 mm), while mortar mixtures: VA-S-25%, VA-S-50%, and VA-S-75% are classified as stiff mortars (with slumps of less than 140 mm). It should be noted that superplasticizer was added to the VA-S-50% and VA-S-75% mixtures to improve their workability, yet they are still considered stiff mortars.

#### 3.3.2. Water Absorption

Durability of mixtures is related to capillary water absorption. In fact, the lower the capillary water absorption, the more durable the mortar is when exposed to environmental agents.

Figure 8 shows the water absorption of the mortar mixes studied. It can be seen that reference series (RM) exhibits the highest absorption of all the mortar mixtures tested. It is important to note that water absorption via immersion in standard test conditions (no vacuum or pressure applied) allows the volume of accessible pores (those that are linked to the surface of the specimen) to be quantified, and pores that are larger than a minimum size are required. Gel and capillary-sized pores (see further details in the analysis in Section 3.3.3), as well as existing pores that are not connected to the specimen surface, are not prone to the effects of water absorption via immersion. Thus, the amount of water absorption determined by this test is substantially inferior to the total porosity quantified by the mercury intrusion method (see Section 3.3.3). Replacing cement with VA leads to a slight reduction in water absorption, although this reduction is negligible. Water absorption appears to be more susceptible to the replacement of sand with VA. In fact, replacing 12.5% of the sand resulted in 7% water absorption, the lowest value observed. As the amount of sand replaced increases, water absorption tends to increase (but always with a value lower than that of the reference mortar). However, the variation between the series with a higher level of water absorption (7.21%) and that with a lower level (7.01%) is not very significant.

**Figure 9:**
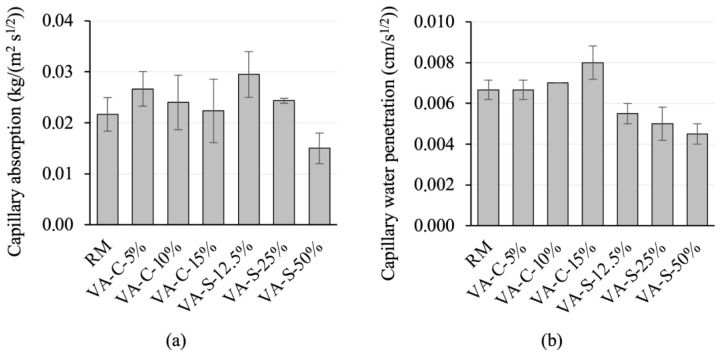
Capillary absorption coefficient (**a**) and water penetration coefficient (**b**) of the mortar mixes.

The capillary water absorption coefficient (AC), and the capillary water penetration coefficient (B) for the mortars studied were determined (see Figure 9). Coefficient AC is the slope of the linear section of the curve obtained by plotting mass variation per area (*Q_i_*) against the square root of time (*t_i_*^1/2^). The capillary water penetration coefficient B is represented by the slope of the curve obtained by plotting the migration height of the water front (*H_i_*) against the square root of time (*t_i_*^1/2^).

Coefficient B does not appear very sensitive to cement being replaced by VA. A slight increase occurs at the 15% replacement level, but the value does not vary significantly before this percentage. However, substituting sand with VA has a greater influence on this parameter. All the series with partial sand replacement have lower B coefficients, and they also have a tendency to decrease as the replacement percentage increases.

The capillary absorption coefficient increases slightly with small cement replacements. As the substitution level increases, this parameter tends to decrease, approaching the value observed in the reference series. In the case of sand substitution, AC is more sensitive to the amount of replacement. This parameter increased significantly at the replacement level of 12.5% when compared to the reference series, and it decreased progressively with higher substitution percentages. The value at the replacement level of 50% is 25% lower than that of the reference series.

Series VA-S-50% had lower capillarity coefficients than the other series. These results are apparently inconsistent with the water absorption results described earlier (see Figure 8). However, the capillarity effect requires the existence of pores whose sizes are within certain limits. Pores with large diameters limit the capillarity effect, but they favor water absorption under immersion. In Section 3.3.3, the porous structures of the series tested are analyzed, and it is concluded that the microstructure of the VA-S-50% series is less refined than the other series. This may explain the seemingly better performance of series VA-S-50% in terms of capillarity.

#### 3.3.3. Porosity

**Figure 10:**
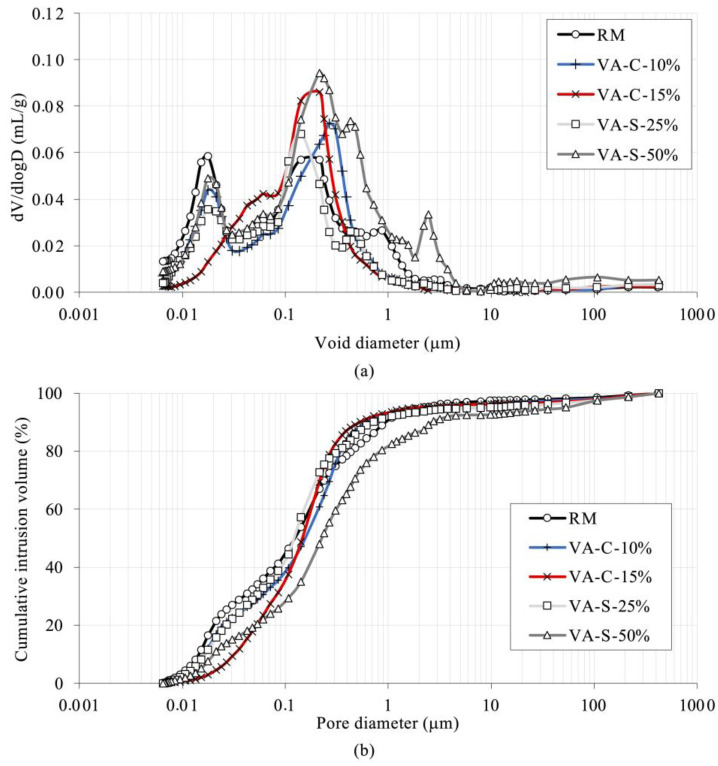
Pore distribution in the mortars tested: pore volume (mL/g) (**a**) and cumulative pore size (%) (**b**).

The mortars under study were subjected to a mercury intrusion porosimetry test to assess their pore size distribution (see Figure 10). Total porosity was quantified, and the results showed that total porosity was quite similar across all the series, except for VA-S-50%. This parameter ranged from 15.7% in VA-S-25% to 17.1% in VA-C-15%. The VA-S-50% series was an exception, exhibiting a total porosity of 24.6%, which is the highest value of the series tested.

Figure 10 shows that the VA-S-50% specimen, which has the highest porosity volume, contains larger pores than the other series, and therefore its microstructure is less refined. The volume of pores with sizes greater than 1 µm in this series is higher than in the other mortars, representing about 17.5% of the total porosity in this series. The pore size distribution for all the other series has a similar pattern, with two distinct peaks: one around 20 nm and the other in the 100–200 nm range (see Figure 10a).

**Figure 11:**
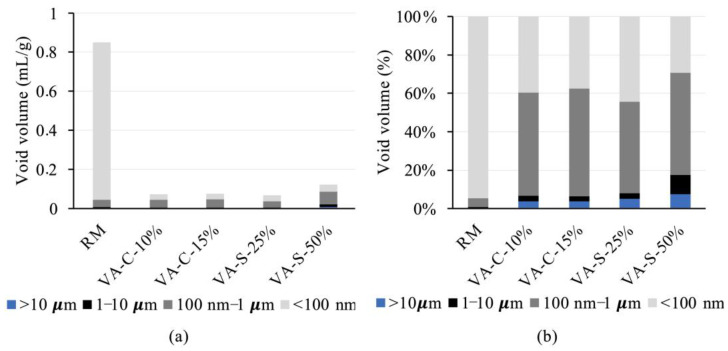
Volume of gel and capillary pores: absolute volume (**a**) and in percentage of total void volume (**b**).

Figure 11a illustrates the volume of gel and capillary pores. Pores with diameters smaller than 100 nm are classified as gel pores, while those ranging from 100 nm to 10 µm are considered capillary pores. As has been reported in several studies [37,38,39,40], gel pores are those with diameters lower than 100 nm, while the pores with sizes in the ranges 100 nm to 1 µm and 1–10 µm are associated with small and large capillary pores, respectively.

The total pore volume in the series tested falls within the range of values reported in the literature for similar materials [39]. The absolute pore volume in the RM series is substantially larger than in the other series. However, a significant percentage of the pores in this series are within the gel pore size range. The VA-S-50% series has the second-highest absolute pore volume, with significantly larger pore sizes than the other series. It is interesting to note the higher porosity of the VA material itself compared to the dolomitic sand used in the mixtures.

In Figure 11b, the pore size distribution as a percentage of total pore volume for all the series tested is shown. The reference series, RM, exhibits the highest percentage of gel and capillary pores of all the series, with 99.7% of the pores falling within these categories. Notably, nearly 95% of the pores in the RM series are within the gel pore range, as previously mentioned.

For the VA-C-10% and VA-C-15% series (where cement is partially substituted by ground ash), the pore size distribution is quite similar. In these series, gel and capillary pores account for 96% of all the pores. However, compared to RM, approximately 56% to 58% of the pores in these series fall into the capillary class, indicating that substitution resulted in an increase in the size of smaller pores, thus leading to a less refined microstructure. This may explain the increase in capillary absorption observed earlier (see Figure 9).

The microstructures of the VA-C-10% and VA-C-15% series are very similar to that of the VA-S-25% series, although the capillary pores in the latter are slightly smaller, comprising 50.8% of the total pores. In contrast, the VA-S-50% series has a less refined microstructure than the other series. Only 29% of its pores are gel pores, and there is a greater proportion of larger capillary pores. This series also shows a noticeable volume (7.5%) of larger pores (>10 µm), which is more than double the percentage observed in the VA-C series (3.8%).

Pore size has a direct influence on the mechanical strength parameters of mortars. While gel pores are too small to initiate cracking, the dimensions of capillary pores are high enough to initiate cracking caused by loading. Moreover, a refined pore structure leads to more homogenous and denser mixtures, with better mechanical properties and durability. A governing factor for absorption depends on the structure of the capillarity of the material: the smaller the capillary network, the lower the absorption of mortar. Accordingly, VA-S-50% is likely to have issues with durability.

### 3.4. Microstructure of the Mortar Mixtures

A SEM investigation was conducted to visualize the change in the microstructure of the mortar responsible for the variation of the physical properties of the samples.

**Figure 12:**
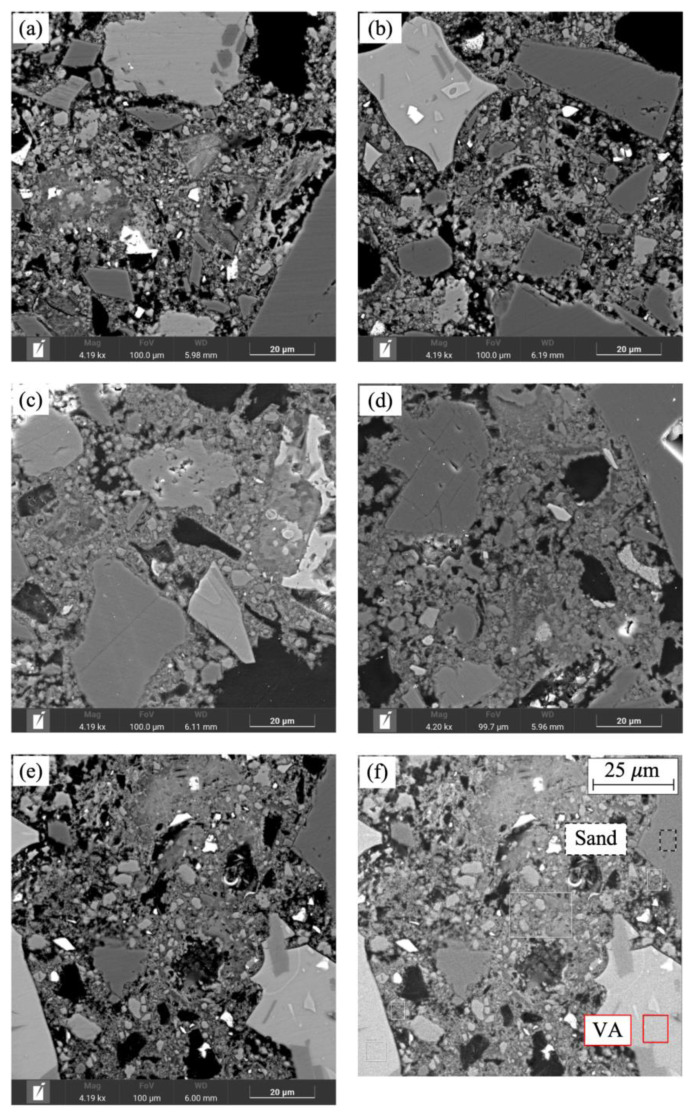
SEM images of specimens (**a**) RM, (**b**) VA-C-10%, (**c**) VA-C-15%, (**d**) VA-S-25%, and (**e**,**f**) VA-S-50%.

Figure 12 illustrates the micrographs of some of the mortar mixtures analyzed with SEM/EDS which was used to examine the structure of the samples. The tests were carried out when the samples were four months old. The black areas correspond to portions of material that were torn off during the sheet cutting process of the SEM specimens. Heavier elements are bright when compared to the dark areas of lighter elements. VA and sand particles are identified in Figure 12f. The introduction of ground VA as a cement replacement led to a homogeneous matrix, similar to RM (see Figure 12b,c). However, specimens in which sand is replaced by VA have more pores (darkest phase) and a more heterogeneus matrix can be observed. A comparison of the micrographs in Figure 12 shows that the specimens with cement replacement are comparatively more compact than those with sand replacement. The greater the level of sand replacement, the less compact, dense, and homogeneous the mortar becomes, which is consistent with a loss of strength and an increase in absorption.

Regarding the paste–aggregate interface in VA, the micrographs show that it is very similar to that of natural aggregates (see Figure 12d,e). The EDS analysis results show that the fundamental elements of the mortar mixtures studied are O, Si, and Ca (see Table 4). This indicates the presence of pozzolanic materials in the mortar matrix that uniformly disperse throughout the matrix. SEM-EDS analysis of mortar mixes revealed the presence of Ca, Al, and Si, suggesting the formation of calcium silicate hydrate (C-S-H) gels derived from OPC, as well as calcium aluminosilicate hydrate (C-A-S-H).

## 4. Conclusions

In this work, the use of volcanic ash from the recent eruption (2021) of the Cumbre Vieja volcano in La Palma (Spain) as a substitute for cement or sand in mortars was studied. Mortar mixtures with different percentages of cement (VA-C series) or sand (VA-S series) replaced by ground VA and VA, respectively, were produced. The influence of VA on the mechanical properties of the mortars was experimentally determined. Based on the results obtained in this research, the following conclusions can be drawn:

1. The workability of the VA-C series was similar to RM, while the VA-S series were classified as stiff mortars.
2. The influence of replacing cement with ground VA on water absorption was negligible. Water absorption appeared to be more sensitive to the replacement of sand with VA, resulting in lower values than RM. A governing factor for absorption depends on the structure of the capillary of the material: the smaller the capillary network, the lower the absorption of the mortar.
3. The capillary absorption coefficient and water penetration coefficient were not significantly affected by the replacement of cement with VA. In contrast, when sand was substituted by VA, it had a greater influence on both parameters. The VA-S series showed a lower water penetration coefficient than RM. The capillary absorption coefficient was highly influenced by the amount of sand replaced by VA. Both coefficients tended to decrease as the amount of VA increases.
4. Total porosity was quite similar across all the series, with the exception of VA-S-50%, which exhibited a significantly higher value than the other series. The microstructures of VA-S-50% mortars were less refined than the other series.
5. The VA-C series showed lower compressive strength after being cured for 28, 90, and 200 days when compared to RM. Conversely, the VA-S-12.5% and VA-S-25% mixtures exhibited higher compressive strengths. VA-S-75% is not recommended because of its low compressive and flexural strengths. A similar pattern was observed with flexural strength. Most of the mixtures showed gained greater flexural and compressive strengths over time than RM when comparing the 28-day results with the 200-day results.
6. Regarding shrinkage, there were no significant deviations in the mixes with maximum replacement levels, VA-C-15% and VA-S-50% mixtures, when compared to the reference mixture (in both air-cured and immersed specimens).
7. Based on the SEM/EDS analysis, the introduction of ground VA as a cement replacement led to a homogeneous matrix, similar to RM. When sand was replaced by VA, the paste-aggregate interface in VA was very similar to that of natural aggregates.

It can be concluded that VA could be suitable as partial replacement for cement or sand in mortars. Based on the results of mechanical properties and durability, it can be concluded that partially replacing cement with up to 15% ground VA as well as substituting sand with up to 25% VA are promising strategies for the production of sustainable mortar mixes.

One of the risks associated with the partial replacement of traditional mortar components by alternative materials is the potential impact on the durability of the new material. Although the current research did not include specific tests to evaluate durability issues, the results suggest little to no impact from the use of VA. In fact, most durability concerns are linked to porosity, which can facilitate the ingress of environmental chemicals such as chlorides, CO_2_, or sulphates into the mortar. The results indicate that the effect of volcanic ash on porosity is minimal, and thus no significant risk to durability is anticipated from this factor. However, some chemical constituents of volcanic ash identified in this study, namely MgO and K_2_O, may potentially promote undesirable internal chemical expansive reactions. Thus, further tests are required to quantify the impact of volcanic ash on mortar durability.

## Figures and Tables

**Table 1:** Chemical composition by XRF of VA, sand, and cement (weight%).

Material	SiO_2_	Al_2_O_3_	Fe_2_O_3_	MnO	MgO	CaO	Na_2_O	K_2_O	TiO_2_	P_2_O_5_	LOI
VA	43.63	13.47	13.23	0.19	8.39	11.27	3.56	1.49	3.63	0.76	0.00
Sand	0.34	0.12	0.06	<LLD	21.46	31.58	<LLD	0.02	0.04	<LLD	46.05
Cement	39.59	14.62	14.91	0.20	6.20	11.36	5.56	1.77	4.20	0.82	0.00

**Table 2:** Dosages of mortar mixtures (quantities indicated in g).

Sample	Sand	Cement	VA	Ground VA	Water
RM	1350	450	0	0	225
VA-C-5%	1350	427.5	0	22.5	225
VA-C-10%	1350	405	0	45	225
VA-C-15%	1350	382.5	0	67.5	225
VA-S-12.5%	1181.3	450	168.75	0	225
VA-S-25%	1012.5	450	337.5	0	225
VA-S-50% *	675	450	675	0	225
VA-S-75% *	337.5	450	1012.5	0	225

* A superplasticizer was added to the mixture to improve workability.

**Table 3:** Bulk density of fresh and hardened mortar mixes in g/cm^3^ and slump flow diameter in mm.

Mix	Bulk Density (g/cm^3^)		Flow (mm)
	Fresh	Hardened	
RM	2.45 ± 0.01	2.44 ± 0.02	163 ± 6
VA-C-5%	2.40 ± 0.05	2.39 ± 0.04	171 ± 4
VA-C-10%	2.38 ± 0.01	2.36 ± 0.03	168 ± 6
VA-C-15%	2.39 ± 0.03	2.38 ± 0.03	166 ± 4
VA-S-12.5%	2.36 ± 0.02	2.35 ± 0.02	153 ± 6
VA-S-25%	2.30 ± 0.01	2.25 ± 0.09	134 ± 10
VA-S-50%	2.18 ± 0.05	2.17 ± 0.04	135 ± 13
VA-S-75%	1.93 ± 0.03	1.92 ± 0.03	105 ± 7

**Table 4:** EDS (wt%) of RM, VA-C-10%, VA-C-15%, VA-S-25%, and VA-S-50% specimens.

Specimen	Element (wt%)							
	O	Mg	Al	Si	S	Cl	Ca	Fe
RM	35.67	1.52	1.26	10.95	1.12	0.17	46.72	2.59
VA-C-10%	35.77	2.45	1.18	10.70	1.16	0.19	46.79	1.76
VA-C-15%	34.80	1.46	0.94	9.44	0.93	-	51.82	0.61
VA-S-25%	33.32	0.78	1.07	6.85	1.14	0.25	54.46	2.13
VA-S-50%	36.31	2.07	2.01	11.69	1.18	-	46.87	0.86

## Data Availability

The data that support the findings of this study are available from the corresponding author, M.A. Fernandez-Ruiz, upon reasonable request.
